# Analyzing Twitter as a Platform for Alzheimer-Related Dementia Awareness: Thematic Analyses of Tweets

**DOI:** 10.2196/11542

**Published:** 2018-12-10

**Authors:** Tiffany Yi-mei Cheng, Lisa Liu, Benjamin KP Woo

**Affiliations:** 1 University of California, Los Angeles Los Angeles, CA United States; 2 Department of Psychiatry University of California, Los Angeles Los Angeles, CA United States

**Keywords:** social media, Twitter, dementia, social support

## Abstract

**Background:**

Dementia is a prevalent disorder among adults and often subjects an individual and his or her family. Social media websites may serve as a platform to raise awareness for dementia and allow researchers to explore health-related data.

**Objective:**

The objective of this study was to utilize Twitter, a social media website, to examine the content and location of tweets containing the keyword “dementia” to better understand the reasons why individuals discuss dementia. We adopted an approach that analyzed user location, user category, and tweet content subcategories to classify large publicly available datasets.

**Methods:**

A total of 398 tweets were collected using the Twitter search application programming interface with the keyword “dementia,” circulated between January and February 2018. Twitter users were categorized into 4 categories: general public, health care field, advocacy organization, and public broadcasting. Tweets posted by “general public” users were further subcategorized into 5 categories: mental health advocate, affected persons, stigmatization, marketing, and other. Placement into the categories was done through thematic analysis.

**Results:**

A total of 398 tweets were written by 359 different screen names from 28 different countries. The largest number of Twitter users were from the United States and the United Kingdom. Within the United States, the largest number of users were from California and Texas. The majority (281/398, 70.6%) of Twitter users were categorized into the “general public” category. Content analysis of tweets from the “general public” category revealed stigmatization (113/281, 40.2%) and mental health advocacy (102/281, 36.3%) as the most common themes. Among tweets from California and Texas, California had more stigmatization tweets, while Texas had more mental health advocacy tweets.

**Conclusions:**

Themes from the content of tweets highlight the mixture of the political climate and the supportive network present on Twitter. The ability to use Twitter to combat stigma and raise awareness of mental health indicates the benefits that can potentially be facilitated via the platform, but negative stigmatizing tweets may interfere with the effectiveness of this social support.

## Introduction

Dementia is a neurocognitive disorder that affects cognitive function and performance of daily activities, such as going to the bathroom, eating, and communicating. In 2017, an estimated 5.5 million people in the United States had Alzheimer-related dementia, one of the most common forms of dementia, with 96% of them being adults aged ≥65 years [1]. The increasing number of adults living with dementia has simultaneously caused an increase in awareness for the mentally debilitating disease. However, this recognition of dementia as a problem among the elderly has caused both negative stigmatization and positive support among communities. For instance, those living with dementia have reported experiencing various degrees of shame, including avoidance, negative self-perceptions, and uncertainty [2]. Interviews with individuals affected by dementia have shown that these individuals often feel perceived as “stupid” [3]. These perceptions of individuals affected by dementia have been perpetuated by the increased use of the internet as a source of information and social commentary. A study examining stigma associated with Alzheimer disease on Twitter found that 21% of Alzheimer-related tweets used related keywords (ie, “Alzheimer’s,” “senile,” “memory loss,” and more) to perpetuate public stigma [4].

Nonetheless, technology has also become a platform for disseminating information about dementia and the creation of education and support programs. This most often occurs in the form of blogs written by dementia caregivers about their experiences and the impact of caring for affected persons. These platforms have aided the development of intervention programs and services for caregivers [5]. Additionally, these platforms have also helped in the creation of these same programs for affected persons. Studies discovered that a dementia awareness campaign may be useful in decreasing mental health disparities [6] while media outlets such as public radio stations would be helpful in promoting dementia awareness [7].

Twitter has been used as a Web-based source for people to receive support [8] and for physicians to share scientific information with the public [9]. Thus, upon analysis of tweets mentioning the term “dementia,” we expect to find supportive Web-based Twitter discussions about dementia. This study is notable for analyzing geotags associated with each tweet to determine the worldwide usage of “dementia” in tweets. As the Western world has become more involved in mental health advocacy, we specifically focus on states within the United States to evaluate whether states that have more open discussions on mental health and mental health policy have more users involved in positive conversations.

The aim of this paper was to develop a better understanding of the Web-based Twitter discussion about dementia and to analyze the applicability of using Twitter as a Web-based support system for individuals with dementia and their families.

## Methods

### Data Collection

A total of 398 publicly available tweets were collected on 4 different dates and times during the months of January and February in 2018 (Table 1). Given the vast majority of tweets available on the Web and the ability of two researchers to manually code each tweet, a sample of 398 tweets was chosen, which is comparable to the 311 tweets manually coded in a similar paper [4]. The Twitter search application programming interface (API) and Postman API Development Environment (San Francisco, CA) were used to collect data endpoints from Twitter that contain the keyword “dementia.” Furthermore, ≤100 tweets were collected at each time point, a limit that is imposed on the Twitter search API. Thus, we accessed only a portion of the tweets posted during each of the 4 time points. For each tweet, we collected data on the date, time, location, username, and tweet body text including hashtags, links, and emojis. Tweets were collected at various time points to account for differences in usage time on the Web and events that may occur, such as political occasions or book publishments.

### Data Analysis and Manual Coding

Upon completion of data collection, all tweets were imported into a password-protected Excel (Microsoft) file for analysis. Retweets were considered as individual data points because the more retweets a particular tweet has, the more likely it is to appear at the top of a Twitter search. Thus, retweets are vital for understanding which category of tweets is more readily visible and available for users searching for “dementia” and the diffusion of tweets through follower-friend connections [10]. Tweets in languages other than English were inputted into Google Translate to identify subject matter.

**Figure 1:**
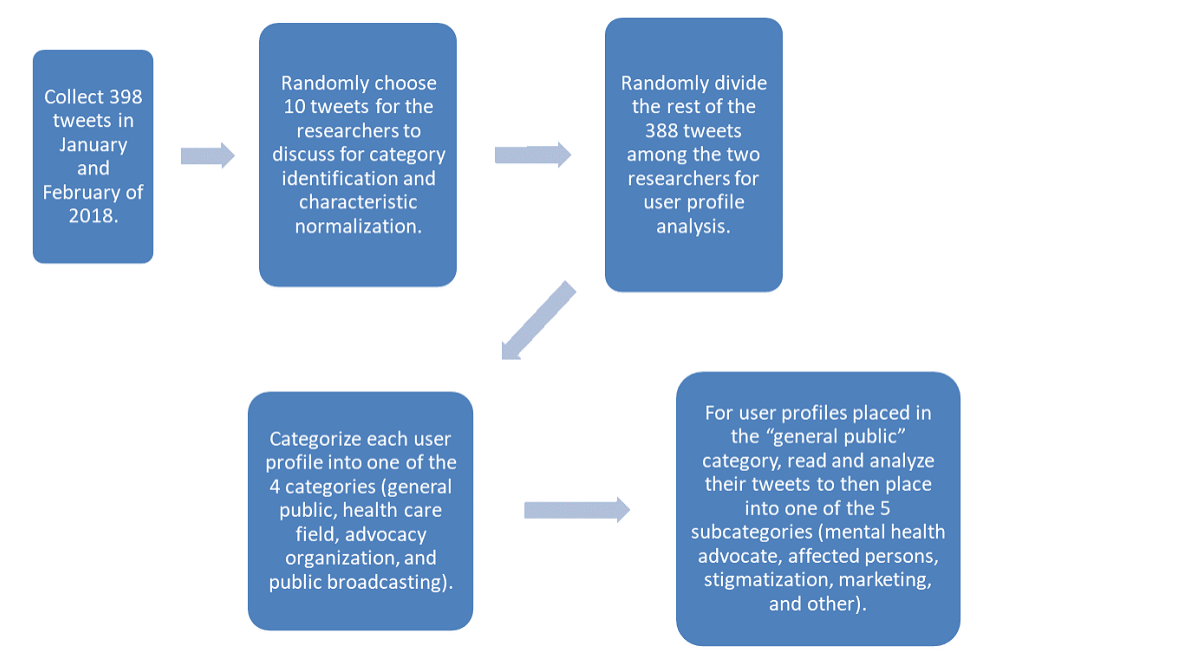
Schematic illustrating the approach to analysis.

User profile information was used to determine the location of the Twitter user. Tweets coming from the United States were further categorized by state. Thematic analysis performed by two of the researchers was utilized to determine the key purpose of each tweet. A few randomly chosen tweets were discussed between the two researchers to determine what category they should be placed in. After tweets from each category were identified and characteristics of each category were discussed, the rest of the tweets were randomly divided to be read by either researcher. Tweets that one researcher was unsure about were then discussed with the other researcher until a category was decided and agreed upon. A hierarchical structure of categories and subcategories was produced to normalize the comparison between tweets. Tweets were divided into 4 categories based on user profile information: general public, health care field, advocacy organization, and public broadcasting. Further classifications were made if the user was considered general public, explained in further detail below. This process is also further illustrated in Figure 1.

**Table 1:** Time points of collection.

Date	Time (Greenwich Mean Time)	Tweets collected (N)
Friday, January 5, 2018	10:12-10:23	100
Thursday, January 11, 2018	6:59-7:16	98
Thursday, February 8, 2018	20:05-20:12	100
Tuesday, February 13, 2018	16:29-16:41	100

For the first category, general public, a user was placed into it if the user was mainly an individual posting about his or her life, thoughts, or interests, and did not have a user description that would place them in any of the other categories. These tweets were subsequently divided into 5 categories based on the content of the tweet itself: mental health advocate (posting about ways to prevent dementia, raising money for dementia), affected persons (persons living with dementia, or family members or friends of a person living with dementia), stigmatization (using the word “dementia” to accuse people of certain qualities), marketing (using dementia to promote a business), and other (not belonging to any of the above named subsections). In the health care field category, the researchers looked for keywords in the users’ profiles that would indicate provider-patient interactions. The users were analyzed for a link to their website on their profile or an explicit statement in the description. For the advocacy organization category, researchers looked for a profile that focused on raising awareness for dementia. Public broadcasting users typically had news-related profiles or usernames. Examples of the users and tweets in the categories can be found in Multimedia Appendix 1. A few words and identifying information have been altered and removed, respectively, to preserve user identity and privacy. This technique has been recommended in prior research on analyzing Twitter data [11].

## Results

### Data Collection

A total of 398 tweets were collected. Of them, 228 (57.3%) were retweets and 359 (90.2%) were posted by different user screen names; furthermore, 29 (7.3%) tweets were posted by the same user screen name at least two or more times.

### Data Analysis and Manual Coding

Upon analysis of geotag locations, tweets were found to be posted from 28 different countries. The top 2 countries with the most tweets were the United States (119/398, 29.9%, tweets) and United Kingdom (111/398, 27.9%, tweets). Of the 119 tweets from the United States, the top 2 states with most tweets were California (22, 18.5%, tweets) and Texas (12, 10.1%, tweets).

Categorization of Twitter users showed that of 398, a majority were classified as general public (281, 70.6%, tweets) and advocacy organizations (57, 14.3%, tweets); moreover, 41 (10.3%) tweets were from users associated with the health care field, such as psychologists, nurses, and researchers, while 14 (3.5%) tweets were associated with public broadcasting, such as newspapers and radio stations. Of the 398 Twitter user profiles, 5 (1.3%) were deleted when analysis began and, thus, could not be classified.

Further analysis of the “general public” category demonstrated that of 281, a majority of the tweets were identified as stigmatization (113, 40.2%, tweets) or mental health advocate (102, 36.3%, tweets); furthermore, 26 (9.3%) tweets were tweeted by affected persons, while 13 (4.6%) tweets were tweeted for marketing purposes. Of 281, 25 (8.9%) tweets could not be classified under the 4 main subcategories and were subsequently placed in the “other” category. A list of keywords used commonly in each subcategory can be found in Multimedia Appendix 2.

Analysis of tweets from the top 2 states in the United States, after adjusting for missing data values, showed that Californians, with 14 tweets, had more stigmatization-related tweets (n=9), while Texans, with 5 tweets, had more mental health advocate-related tweets (n=3).

## Discussion

### Principal Findings

Less than a third of the tweets were posted by advocacy organizations or health professionals. These tweets generally aim to raise awareness about dementia, provide dementia prevention information, and give advice for caretakers. However, many of these tweets are eclipsed by the many more tweets generated by the general public. Web users seeking support from dementia tweets may not see all of the information offered to them by advocacy organizations or health professionals but instead see information provided by the general public. This leads to dangers of misinformation through the Web as many are willing to believe news that align with their own beliefs and provide them with a greater sense of hope and control [12]. While many of the posts from the general public may be helpful, posts from organizations or professionals are more likely to contain credible and trustworthy information that will be better received and followed by the public.

Furthermore, many of the tweets posted by the general public in the “mental health advocate” and “affected persons” subcategories provide sentimental tweets that raise awareness and seek support, respectively. Having these become eclipsed by negative tweets reduces the effectiveness of using Twitter as a supportive Web-based community for dementia.

In 2009, the National Alliance on Mental Illness produced a report card for each state, grading them in 4 categories related to their mental health policies [13]. This standard was utilized to compare the mental health system in both California and Texas (Table 2). Because California has a higher grade in each category, it demonstrates that California has more resources and ability to advocate for those living with mental illnesses. Thus, we would expect a larger amount of mental health advocates among Twitter users residing in California rather than in Texas. However, results demonstrate that there are more stigmatization posts from Twitter users residing in California than those in Texas. This contradicts what is expected based on the grades given to each state.

### Limitations

In exploring the global conversation surrounding dementia, a majority of the tweets were posted from the United States and United Kingdom. Although the United States and United Kingdom are among the top countries that have the most Twitter users, there are other countries such as Japan and Spain that may engage in the dementia conversation [14]. Since the search term used was “dementia” in English, this may have limited the searched tweets to those written in English. Furthermore, the United States and United Kingdom are often at the forefront of increasing awareness for mental health awareness. As one of the first countries to establish a mental health policy in 1996, the United States has seen its residents hold increasingly positive attitudes toward seeking professional help for mental health problems over time [15]. Another factor to consider is the role of culture in mental health awareness and seeking professional help [16]. This may influence the lack of other countries actively participating in the dementia discussion.

In relation to the deviance from data suggesting that California would generally have more mental health advocates than Texas, data collected during the month of January followed large political events or wrongdoings. Thus, Twitter users might have used the term “dementia” to express negative attitudes toward those involved in the political event.

### Comparison to Prior Works

A similar study on Twitter that was performed in 2012 studying types of social media users and dementia themes found that most of the information on Twitter came from health professionals, health information sites, new organizations, and commercial entities and that most tweets contained links to news and health information sites [17]. From 2012, the number of worldwide active Twitter users has greatly increased from 167 million users to 335 million users in 2018 [18]. Now that there are more users, there can be more actively voiced, differing opinions and misinformation that may affect how others perceive dementia and bar access to dementia support systems. Thus, as the world uses more technology, it becomes more relevant and important to study how the Web-based Twitter discussion may have changed over time.

**Table 2:** The 2009 National Alliance on Mental Illness category grades.

Category^a^	California grade	Texas grade
I. Health Promotion & Measurement	B	F
II. Financing & Core Treatment/Recovery Services	C	D
III. Consumer and Family Empowerment	D	F
IV. Community Integration & Social Inclusion	B	D

^a^National Alliance on Mental Illness category grades are based on 4 categories: Health Promotion and Measurement considers the services states provide, the development of mental health policy planning, and data collection; Financing and Core Treatment/Recovery Services entails the accessibility and availability of mental health services, reimbursement for these services through state Medicaid programs, the severity of current shortages in the mental health workforce, and state efforts to improve the cultural competence of their mental health care systems; Consumer and Family Empowerment grades are determined by the states’ opportunities for family and consumer education and empowerment; and Community Integration and Social Inclusion determines whether states are compensating for extreme mental illnesses that go beyond those addressed by mental health agencies (this includes data on public health education and housing resources).

### Clinical Implications

As media outlets have played an important role in communicating popular opinion in the general public [19], researchers have further shown that the Web-based community shows a greater preference for talking about mental health conditions compared with nonpsychiatric diseases, such as cancer, stroke, or HIV infection [20]. Tweeting about mental health can foster communication among the Twitter community [21], especially anonymous communication that allows individuals to express themselves openly and honestly [22]. Twitter surveillance of these discussions may help health care providers, health institutions, and policy makers see how dementia is discussed and what issues are there in their own communities, allowing them to tailor their services to local, current issues presented in Web-based culture of dementia [23].

Furthermore, while platforms such as Twitter can allow for access to pertinent, critical information, medical misinformation on the Web may lead to potential rifts in patient-health care provider relationships. Web-based information that differs from that provided by health care providers can lead to unanticipated consequences, such as raising false hopes or increasing patient anxiety. To combat distribution of misinformation, especially on a large social media platform, health care providers should actively address and recommend websites with information that they deem is qualitatively informative and accurate. [24] This would encourage patients to ignore information on popular social media that is more likely filled with opinionated statements than factual evidence.

### Conclusion

Twitter as a social media platform has great potential for disseminating information on care, creating support systems, and raising awareness for Alzheimer-related dementia. With the popularity of the internet growing with each generation, future generations may turn to Twitter to seek comfort and knowledge about the onset of this disease. However, it has thus far been used as a readily available method for perpetuating stigma by attributing traits associated with dementia to normal, healthy people that Twitter users may find disagreeable.

Further research is warranted to determine the full impact and number of tweets addressing dementia in a negative light or as a method to raise awareness. In particular, older generations that may actually be affected by dementia do not use the internet as a platform for discussion as much as younger generations do. Thus, research on dementia on social media platforms should also be pursued in the future as younger generations grow older. The search for tweets would also have to be broadened to be more inclusive in addressing language differences in tweets (“dementia” in different languages), the time of day the tweets were posted, and the time of year. Furthermore, in the efforts to raise awareness of mental health issues, the conversations surrounding dementia should be expanded from the United States and United Kingdom, changing cultural beliefs that affect views on mental health. With more than 330 million users on Twitter, understanding the ability of Twitter as a readily available, popular, free source of information to better disseminate important information about Alzheimer-related dementia may be the key in building supportive and knowledgeable communities for the future.

## Appendix

Multimedia Appendix 1 Examples of Twitter users and Twitter posts.

Multimedia Appendix 2 List of key words used common in the different types of posts in the general public category.

## Abbreviations

API: application programming interface
