# Supplementary material for: Analyzing Twitter as a Platform for Alzheimer-Related Dementia Awareness: Thematic Analyses of Tweets
Source: JMIR Aging. 2018 Dec 10;1(2):e11542. doi: 10.2196/11542 (PMC6715397; doi:10.2196/11542)
Supplement: Multimedia Appendix 1 [file aging_v1i2e11542_app1.pdf]

Appendix 1

Examples of Twitter users and Twitter posts.

Types of Users	Description
Healthcare field user	Radiology. Administrator. Opinions are my own.
Advocacy organization user	Learning and sharing information to support caregivers and loved ones with dementia.
General Public	Former Blogger | Animal lover | Author
Public Broadcasting	Florida’s Rock Station, where you can listen to rock music! Streaming live 24/7.

Types of Posts	Text
Mental health advocate post	“Even when you have Alzheimer’s disease, you need ways to participate in our community” Dr. Allison.
Stigmatization post	Go to hell, Mr. P. You’re the only one with dementia that we know.
Affected person	Seeing someone you love suffer from dementia is very heartbreaking…
Marketing	C.K’s new fiction novel wins major prizes #dementia
Other	In certain countries, the justice system makes dementia tests for older inmates.
