# Supplementary material for: Analyzing Twitter as a Platform for Alzheimer-Related Dementia Awareness: Thematic Analyses of Tweets
Source: JMIR Aging. 2018 Dec 10;1(2):e11542. doi: 10.2196/11542 (PMC6715397; doi:10.2196/11542)
Supplement: Multimedia Appendix 2 [file aging_v1i2e11542_app2.pdf]

Appendix 2

List of key words used common in the different types of posts in the general public category.

Stigmatization	Names of political figures<br>Derogatory Words<br>Forget<br>Offended<br>Nickname<br>A sign of dementia<br>Accuse<br>Denied<br>Clown
Affected person	Visiting a loved one<br>Seeing a loved one<br>My grandma/grandpa/mom/dad<br>Friend<br>Lost<br>Passed away<br>#diversealz (a hashtag started by an advocacy organization for posts about dementia experiences)
Marketing	Song artist/book author names<br>For dementia patients<br>New technology
Mental health advocate	Suffer from dementia<br>Dementia community/ village/ friends<br>Families affected by dementia<br>Donate<br>Improve<br>Involve/participate<br>Inspired<br>Planning<br>Reduce risk/ prevention/ help<br>Tips
